# Facial Nodular Melanoma Presenting as an Invasive Fungal Infection: A Diagnostic Challenge

**DOI:** 10.7759/cureus.90668

**Published:** 2025-08-21

**Authors:** Chloe R Schmidt, Zachary Smith, Lauren Workman, Vickie Xin, Francisco Davila, Jean Llenos

**Affiliations:** 1 Internal Medicine, Oakland University William Beaumont School of Medicine, Rochester, USA; 2 Internal Medicine, Corewell Health William Beaumont University Hospital, Royal Oak, USA; 3 Pathology, Corewell Health William Beaumont University Hospital, Royal Oak, USA

**Keywords:** facial melanoma, invasive fungal mimic, nodular melanoma, soft tissue mass, sox10 positive tumor

## Abstract

Nodular melanoma is an aggressive subtype of malignant melanoma, accounting for a disproportionate number of melanoma-related deaths due to its rapid progression and early metastatic potential. Despite its distinct histopathological characteristics, clinical diagnosis can be challenging, particularly when the presentation mimics other conditions. We present the case of a 67-year-old male whose facial nodular melanoma was initially treated as an invasive fungal infection due to overlapping clinical and radiographic features, including osseous destruction, soft tissue invasion, and systemic involvement. The patient underwent antifungal therapy, which resulted in acute kidney injury from amphotericin B, prior to a definitive diagnosis by histopathological analysis. This case highlights the importance of maintaining a broad differential diagnosis for atypical facial lesions, especially those with aggressive features or poor response to initial therapies. Early biopsy and tissue evaluation are essential to distinguish malignant melanoma from infectious or inflammatory conditions and to ensure timely, appropriate management.

## Introduction

Melanoma is an aggressive skin cancer known for its ability to spread to distant organs. Malignant melanoma has an incidence rate of approximately 0.9 per 100,000 and carries a poor prognosis [1]. The likelihood of metastasis increases with greater depth and ulceration of the primary lesion, and due to its aggressive nature, melanoma often metastasizes before a diagnosis is confirmed. 

Nodular melanoma, a subtype of malignant melanoma characterized by vertical growth, is particularly aggressive and has a high mortality rate. Although it accounts for only 15% of invasive melanomas, it is responsible for 40% of melanoma-related deaths, primarily due to its rapid mitotic rate, early metastasis, and frequent resemblance to benign conditions. It typically occurs in middle-aged to older adults, especially men with fair skin [2]. Patients often present with a large, asymmetrical lesion that may itch, bleed, or ulcerate. Unfortunately, nodular melanoma frequently metastasizes before diagnosis, presenting considerable therapeutic challenges and poor prognosis [2,3]. Newer therapeutic approaches, such as immunotherapy and targeted therapy, have improved the survival rate of melanoma, particularly if started soon after the initial diagnosis [4].

Invasive fungal infections of the face, such as mucormycosis and invasive aspergillosis, often present with rapidly progressive tissue necrosis, facial swelling, black eschar formation, sinus pain, and ophthalmoplegia [5]. The worrisome nature of these infections stems from their aggressive angioinvasive behavior, which leads to vascular thrombosis, tissue ischemia, and potential extension into the orbit and brain, causing life-threatening complications like cavernous sinus thrombosis and meningitis [6]. Early recognition and urgent intervention with antifungal therapy and surgical debridement are critical to improving survival, as delays can result in extensive tissue destruction and high mortality rates [7].

We describe a rare presentation of nodular melanoma that mimicked an invasive fungal infection, further complicating and delaying the diagnosis. This rare mimicry between nodular melanoma and invasive fungal infection is not well-documented, with few case reports and limited research on this unusual presentation. Increasing awareness of this potential mimicry can help to broaden the differential when confronted with atypical skin lesions. 

This article was previously presented as a meeting abstract at the 2024 MI-ACP and MI-SHM Annual Scientific Meeting on October 24, 2024.

## Case presentation

**Figure 1:**
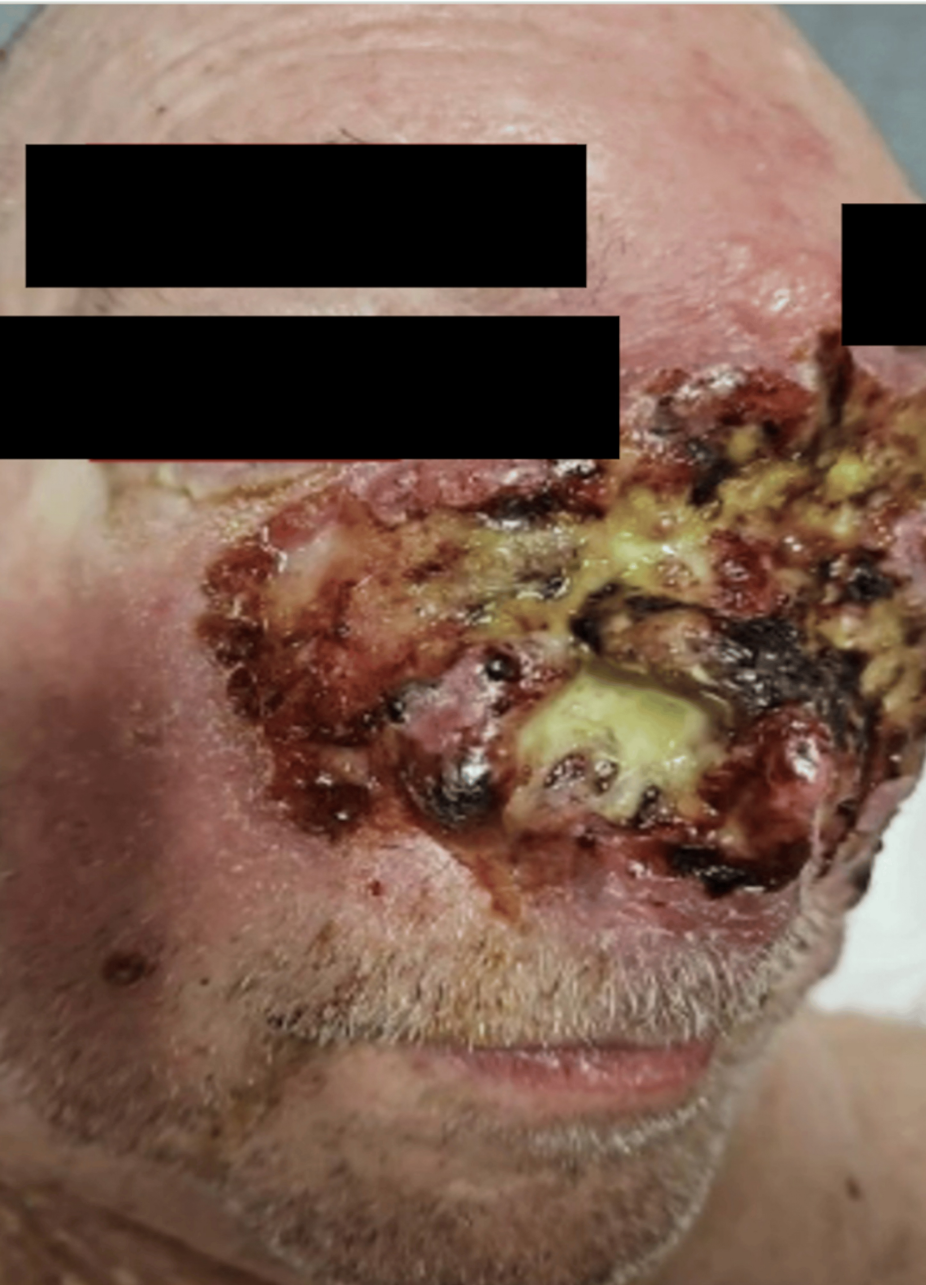
Extensive ulcerated and necrotic facial lesion involving the left periorbital region, cheek, and nasal bridge. The lesion exhibits a mix of necrotic tissue, purulent exudate, and areas of hemorrhage, with surrounding erythema and edema.

A 67-year-old male with past medical history of hypertension and right lower extremity deep vein thrombosis presented with complaints of anosmia and a purulent facial mass that had slowly increased in size over the past four months. He also complained of bilateral leg swelling. No fever, chills, chest pain, or shortness of breath. No weight loss. On presentation, his vital signs were stable, and physical exam demonstrated an ulcerative mass with complete breakdown of the nasal septum (Figure 1) and bilateral non-pitting edema.

**Figure 2:**
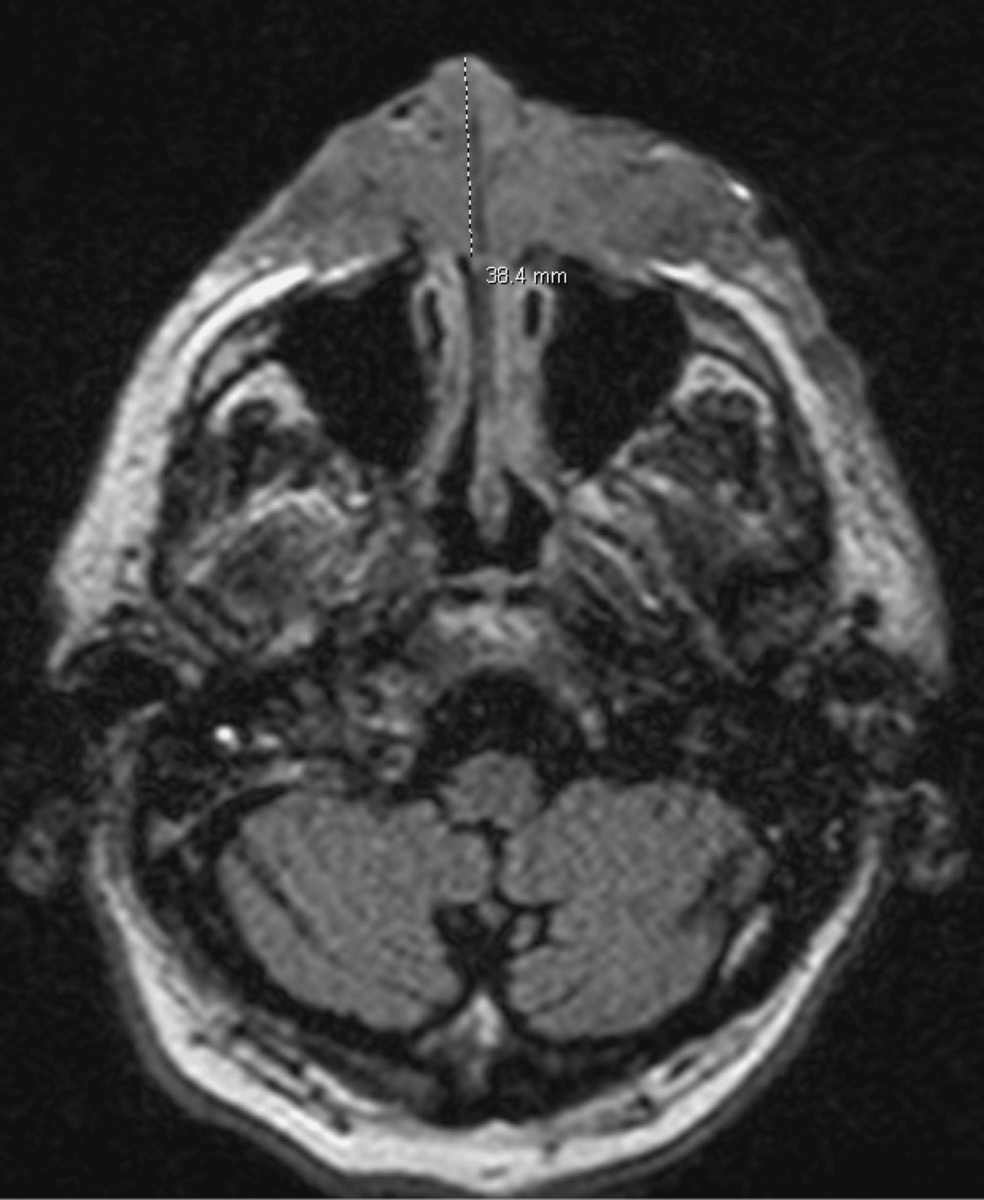
Computer tomography demonstrating a large facial mass measuring 10×10×3.8 cm extending into the upper lip, preseptal orbits, supraorbital frontal scalp, and lateral orbital margins.

Computer tomography demonstrated a large facial mass measuring 10×10×3.8 cm extending into the upper lip, preseptal orbits, supraorbital frontal scalp, and lateral orbital margins (Figure 2).

**Figure 3:**
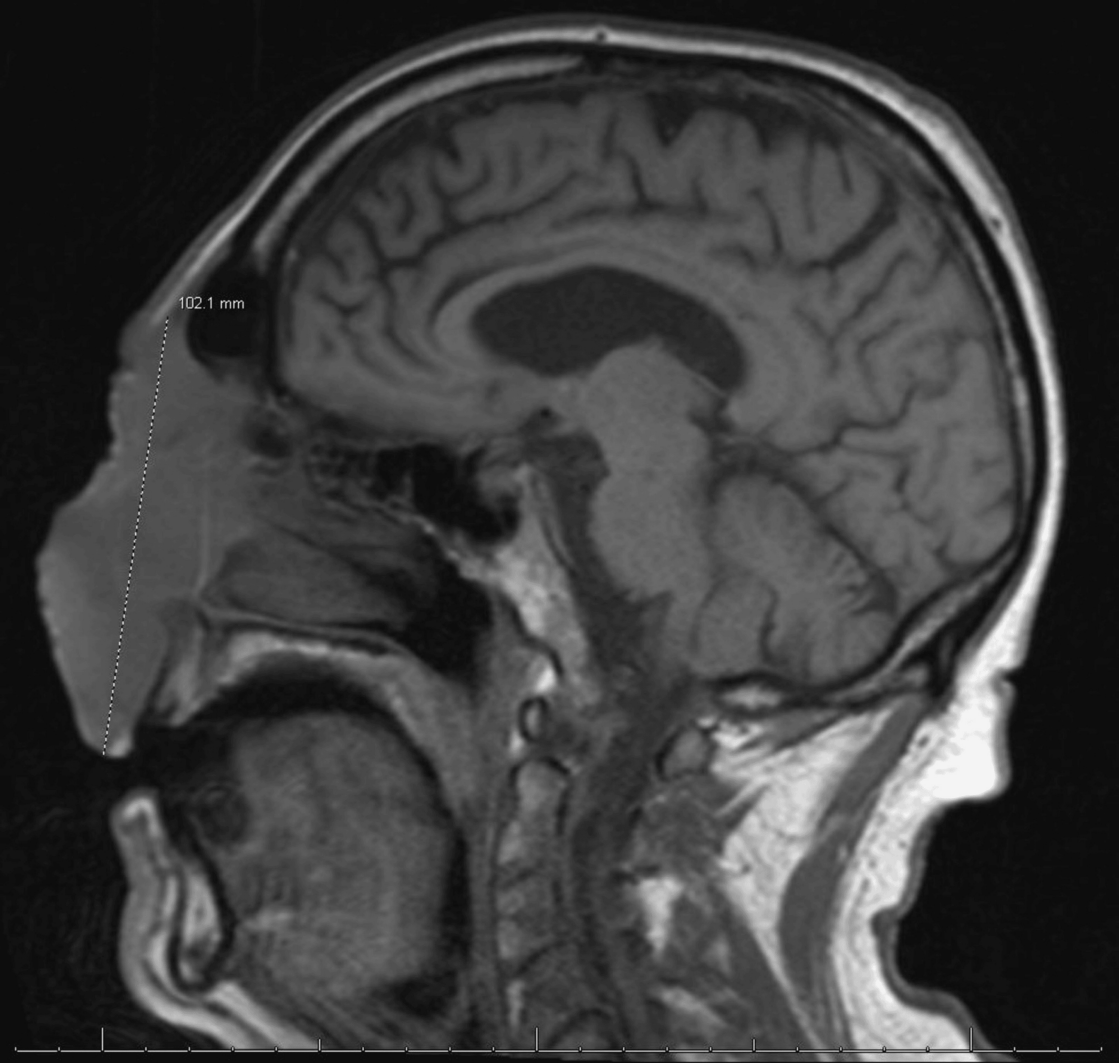
Magnetic resonance imaging (sagittal view) demonstrating osseous erosion of the nasal bone and anterior nasal septum extending posteriorly to the choana on both sides of the nasal septum.

Magnetic resonance imaging demonstrated osseous erosion of the nasal bone and anterior nasal septum extending posteriorly to the choana on both sides of the nasal septum (Figure 3).

**Figure 4:**
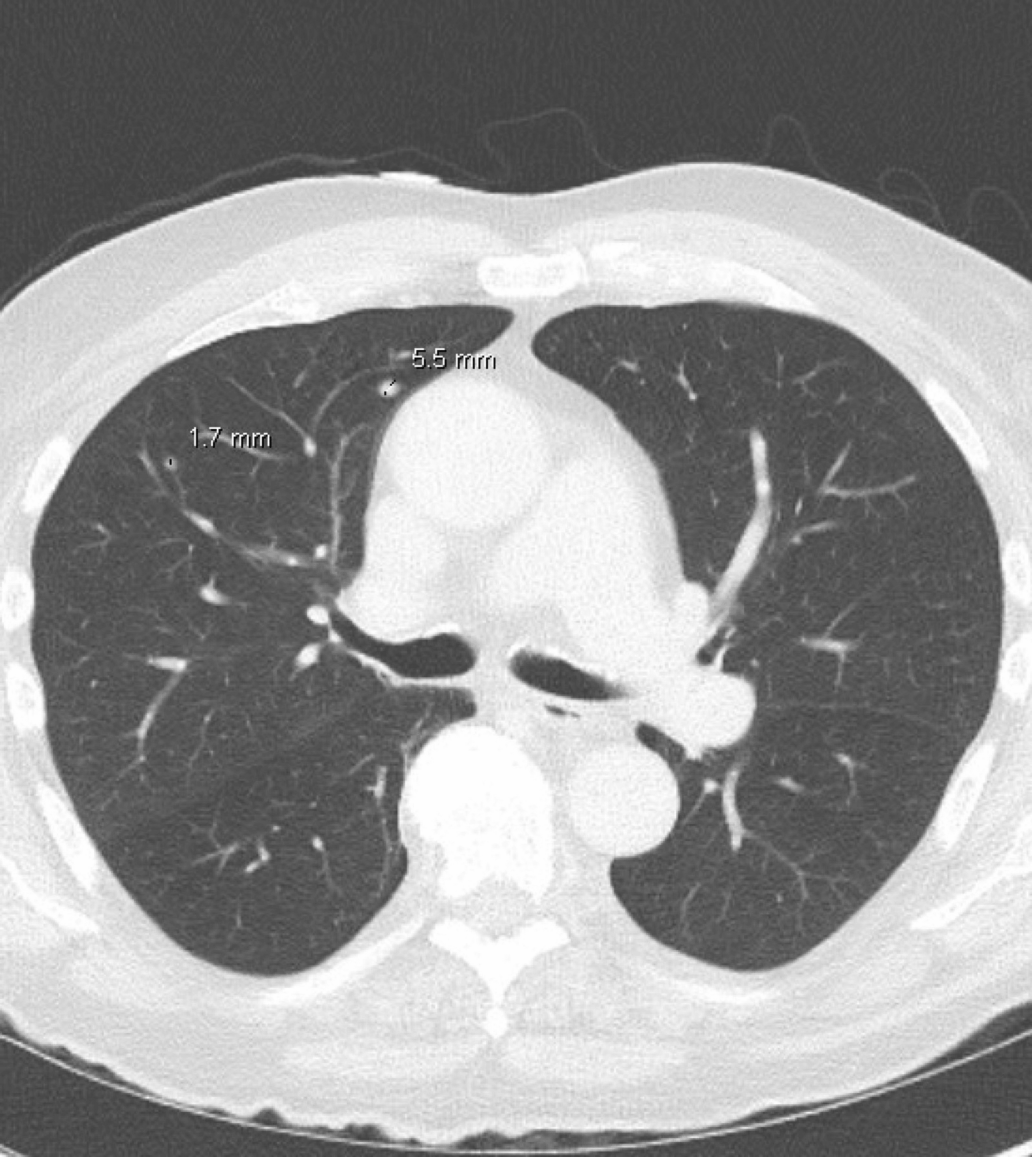
Axial contrast-enhanced CT scan of the chest in lung window demonstrates two non-calcified pulmonary nodules in the right lung measuring 5.5 mm and 1.7 mm.

Further imaging demonstrated cervical lymphadenopathy and several pulmonary nodules concerning for metastatic foci (Figure 4) as well as bilateral lower extremity deep venous thromboses (DVTs).

**Figure 5:**
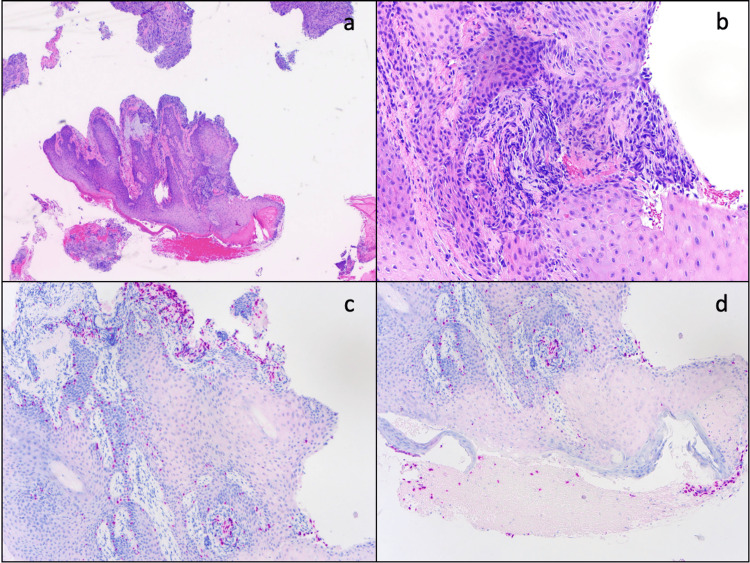
(a) 1.4×-Population of spindled cells demonstrating pleomorphism and desmoplasia within the dermal and epidermal layer. Similar clusters of spindled cells can be seen at the outer edges of the image. (b) 1.20×-20× magnification of the former. (c) 10× magnification of main tissue foci in previous image focusing on top/dermal layer. (d) 10× magnification of main tissue foci in previous image focusing on bottom/epidermal layer.

Laboratory studies demonstrated leukocytosis without eosinophilia, normal creatinine, glucose levels, and hemoglobin A1c. The patient was started on intravenous heparin for the DVTs and vancomycin plus piperacillin-tazobactam for concerns of superimposed infection. Liposomal amphotericin was also initiated due to concern of invasive fungal infection given bony erosion, metastatic foci, and nodules adjacent to the mass. Anticoagulation caused the mass to become friable and bleed, which caused significant discomfort but did not result in anemia. Four days after admission, the patient developed an acute kidney injury (likely from exposure to amphotericin), and blood cultures drawn on arrival returned negative, so this medication was discontinued (Table 1). His creatinine peaked on day six before slowly trending down. Facial biopsy results were not available until day nine, at which point punch biopsies of the facial mass returned negative for fungal hyphae but demonstrated SOX10-positive spindle cell tumor, favoring melanoma (Figure 5).

**Table 1:** Table of laboratory values pre-amphotericin and post-amphotericin. BUN: blood urea nitrogen.

	Pre-amphotericin	Post-amphotericin	Reference ranges
Na^+^	136 mmol/L	139 mmol/L	135-145 mmol/L
K^+^	4.1 mmol/L	3.3 mmol/L	3.6-5.5 mmol/L
Cl^-^	103 mmol/L	108 mmol/L	97-105 mmol/L
HCO3^-^	20 mmol/L	17 mmol/L	22-29 mmol/L
BUN	12 mg/dL	19 mg/dL	6-24 mg/dL
Cr	0.76 mg/dL	3.51 mg/dL	0.7-1.3 mg/dL
Glucose	104 mg/dL	116 mg/dL	70-179 mg/dL
Mg^2+^	1.8 mg/dL	1.8 mg/dL	1.7-2.5 mg/dL
WBC	10.8 bil/L	8.3 bil/L	3.5-10.1 bil/L
RBC	4.21 tril/L	3.66 tril/L	4.31- 5.48 tril/L
Hb	12.6 g/dL	10.8 g/dL	13.5-17.0 g/dL
Hct	39.50%	34.50%	40.1-50.1%

The patient was bridged to warfarin for his DVTs and discharged with plans for outpatient immunotherapy and potential reconstructive surgery. He was initiated on an immunotherapy regimen of nivolumab and ipilimumab shortly after discharge; however, he was subsequently lost to follow-up after six months of immunotherapy, and his long-term clinical status, including disease progression and treatment response, could not be assessed.

## Discussion

Malignant melanoma is a highly aggressive form of skin cancer arising from melanocytes and known for its ability to present with a broad spectrum of clinical and histopathological features, leading to diagnostic challenges [8]. Primarily demonstrated, in this case, is its ability to mimic features similar to invasive fungal infections. Misidentification of malignant melanoma for invasive fungal infection can occur due to several overlapping features. Clinically, both conditions may present with similar skin lesions, including nodules, plaques, and ulcerations [9-11]. Additionally, systemic symptoms such as fever, weight loss, and malaise may be present in both conditions, further complicating the diagnosis. Furthermore, in this case, the initial radiologic impression was heavily influenced by findings commonly associated with invasive fungal infections, particularly angioinvasive mycoses. Imaging revealed a large, destructive facial mass with osseous erosion (Figure 3), soft tissue infiltration (Figure 2), and distant pulmonary nodules (Figure 4), which are hallmark features of angioinvasive fungal infections like mucormycosis and aspergillosis in immunocompromised hosts. Osseous destruction and contiguous spread into the orbits and sinuses raise suspicion for fungal invasion due to their propensity for vascular invasion and rapid tissue necrosis. Additionally, pulmonary nodules in this context may be interpreted as hematogenous fungal dissemination. These overlapping features contributed to the initial working diagnosis of an invasive fungal process and underscore the diagnostic challenge in differentiating aggressive infections from malignancy without tissue confirmation. Thus, it is imperative to differentiate the two. 

Histopathologically, melanoma cells may exhibit significant pleomorphism, mitotic activity, and infiltration into surrounding tissues [12,13]. Invasive fungal infections, on the other hand, typically present with granulomatous inflammation and necrosis [14]. However, fungal hyphae or yeast forms can be mistaken for atypical melanocytes, especially in cases where fungal elements are sparsely or poorly identified [14]. There are several previous cases in the literature of misdiagnosis of melanoma for fungal infection and the resulting consequences of a delayed diagnosis. Kim et al. reported cases of melanoma misdiagnosed as fungal foot infections [15]. Sondermann et al. demonstrated malignant melanoma being mistaken for onychomycosis in a fingernail [16]. Additionally, Chatterjee et al. presented a case of a patient with history of a renal transplant misdiagnosed with cutaneous chromoblastomycosis [17].

In addition to the histopathological differences, the timing of presenting symptoms may also help differentiate the two. Invasive fungal infections are typically rapidly fatal, and patients often feel very ill, presenting with many symptoms such as fever, cough, nasal discharge, and mental status changes [18]. In addition, these infections typically affect immunocompromised individuals, such as those with uncontrolled diabetes, prolonged corticosteroid use, or neutropenia [5]. On the other hand, malignant melanoma typically presents with more indolent symptoms in addition to the appearance of the lesion. Systemic symptoms may only start to develop in advanced cases when the melanoma has metastasized [19]. Our patient presented with minimal systemic symptoms besides those related to the obstructive nature of the mass and leg swelling. The mass itself had been growing for several months, a progression more characteristic of malignancy than of an invasive fungal infection.

In our case, several subtle clinical red flags favored a diagnosis of malignancy rather than infection at the time of initial presentation. First, the chronicity of the lesion, enlarging over several months without the acute deterioration characteristic of invasive fungal disease, was inconsistent with the rapidly progressive nature of mucormycosis or aspergillosis. Second, the patient was not immunocompromised, diabetic, or neutropenic, which are risk factors strongly associated with angioinvasive fungal infections. Third, the lack of significant inflammatory markers or fever further reduced the likelihood of an acute infectious etiology. Lastly, the extensive yet well-circumscribed nature of the mass with metastatic pulmonary nodules could be more in line with an aggressive malignancy than a disseminated fungal infection, especially in the absence of microbiological evidence.

The therapeutic approach for malignant melanoma versus invasive fungal infections is markedly different and both come with unique adverse side effects, underscoring the importance of accurate diagnosis. Malignant melanoma treatment often involves surgical excision with clear margins, adjuvant therapies such as immunotherapy (e.g., checkpoint inhibitors), targeted therapy (e.g., BRAF inhibitors), and in some cases, radiation and chemotherapy [8]. Conversely, invasive fungal infections require antifungal therapy, which may include systemic agents like amphotericin B, azoles, or echinocandins, and sometimes surgical debridement of infected tissues [18]. In this case, the initial suspicion of an invasive fungal infection led to the administration of liposomal amphotericin B, which led to an acute kidney injury in the patient.

Despite the instructive value of this case, it is important to recognize its limitations. As a single case report, the generalizability of its findings is inherently restricted. While it highlights a rare and diagnostically challenging mimicry between nodular melanoma and invasive fungal infection, such presentations may not be common enough to warrant broad diagnostic shifts without further supporting evidence. Future research or case series may help clarify whether specific clinical or demographic patterns can reliably distinguish between these two mimicking entities earlier in the diagnostic process.

Another limitation of this report is the absence of long-term outcome data, as the patient was lost to follow-up after six months of immunotherapy. Consequently, his response to treatment, overall prognosis, and any therapy-related complications remain unknown, which restricts our ability to comment on long-term clinical outcomes beyond six months. 

## Conclusions

This case illustrates the critical need for a high index of suspicion and thorough diagnostic evaluation when confronted with atypical presentations of skin lesions. Malignant melanoma's ability to mimic invasive fungal infections underscores the importance of considering a broad differential diagnosis, particularly in cases where initial treatment does not yield the expected response. Early tissue biopsy should be prioritized in any patient with a chronic or slowly progressive lesion that lacks typical infectious symptoms, especially when the patient is not immunocompromised. In addition, multidisciplinary consultation should be considered early in cases with ambiguous findings as an accurate diagnosis is paramount to initiating the correct therapeutic regimen and avoiding unwanted side effects. While this case contributes valuable insight into the diagnostic challenges of melanoma masquerading as infection, its findings must be interpreted within the context of a single-patient report. The unique presentation and absence of certain immunologic data limit the broader applicability of conclusions. Nonetheless, it reinforces the importance of recognizing the potential for malignancies to present in atypical ways and the value of a multidisciplinary approach and adaptive clinical reasoning in complex cases. 
